# Bayesian LSTM-Based Missing Data Estimation and Flexural Strength Assessment for Determination of Novel Smart Mobility Pavement Materials: Mg(OH)_2_ Added Plastic Composites

**DOI:** 10.3390/ma18184334

**Published:** 2025-09-16

**Authors:** Byeong-Hun Woo, Yong-Joo Kim, Il-Hwan Kang, Kang-Hun Lee

**Affiliations:** Korea Institute of Civil Engineering and Building Technologies, Department of Highway & Transportation Research, 283, Goyang-daero, Ilsanseo-gu, Goyang-si 10223, Republic of Korea; bhwoo@kict.re.kr (B.-H.W.); yongjook@kict.re.kr (Y.-J.K.); kih0223@kict.re.kr (I.-H.K.)

**Keywords:** Bayesian LSTM, pavement, plastic, machine learning, missing data

## Abstract

The construction field needs to reduce carbon emissions; therefore, many methods are being attempted applying to materials research. Concrete and asphalt are the representative materials for the pavement system. However, a lack of aggregates and certain limits in binder replacement are the main obstacles to achieving a reduction in carbon emissions. This study aims to address the lack of sustainable alternatives in pavement materials by investigating recycled plastic composites with Mg(OH)_2_, thereby filling a research gap in low-carbon, mechanically viable solutions for smart mobility infrastructure. Three types of recycled plastics—polypropylene (PP), polyethylene terephthalate (PET), and OTHER (OTH) resins—were combined with Mg(OH)_2_ to produce nine specimen configurations. The mechanical behavior and flexural performance were evaluated through displacement–flexural stress curve tests, while missing experimental data were reconstructed using a Bayesian long short-term memory (BLSTM) machine learning approach. The BLSTM model achieved an average R^2^ of 0.8018 in testing and 0.7618 in validation, confirming reliable prediction capability even with a small dataset. All composites demonstrated a minimum flexural strength of 30 MPa, with PP-based specimens reaching approximately 40 MPa, confirming their suitability for pavement applications. These results highlight the flexural performance of each composite type, with PP emerging as the most promising candidate.

## 1. Introduction

The world now cannot make road pavement without asphalt and concrete. Because of the superiority of asphalt and concrete, the world is using them to pave the way without exception. There are some special conditions necessary for applying other materials to pave the road, such as using geopolymers experimentally [1] and gravels in some conditions [2]. However, most pavements use asphalt and concrete.

Recently, the pavement industry has faced embarrassing problems. The first issue is the lack of natural aggregates. In asphalt concrete pavement, aggregates occupy 90–95% volume fraction [3], and occupy approximately 65–75% volume fraction in the cement concrete matrix [4,5]. Already, a tremendous amount of aggregates have been applied to the construction field; natural aggregates are almost depleted. Therefore, recycled [6] or artificial aggregates [7] have been considered to replace natural aggregates. However, recycled aggregates are collected from demolished structures or end-of-life concrete [6,8]. In this state, recycled aggregates may have damage in their matrix already, which results in higher porosity and reduces the quality of concrete or asphalt materials [9]. Although recycled aggregates are inevitably used, it is clear that the overall quality of concrete or asphalt is deteriorating [10]. On the other hand, artificial aggregates are supplied by factories because they should be manufactured by production systems. Artificially manufactured aggregates usually require high costs [11] because the producing process includes the price of facilities, energy usage, human force, and so on. Hence, the application to the construction field of artificial aggregates has limits due to the mentioned problem.

The second issue is environmental problems. Carbon emission in the cement industry accounts for approximately 8% of global greenhouse gas emissions [12], which is the second largest value. In addition, asphalt pavement systems are also emitting a large portion of greenhouse gas—approximately 1.8% in the specific case of Canada [13]. In addition, hot-melting asphalt needs a high temperature of approximately 150–180 °C, which makes the area hotter. The global warming crisis is steadily growing; the field of construction should find a way to solve this problem. The most representative considered method is not using conventional materials such as cement and asphalt. Already, many studies have tried to prove that alternative materials have potential for application in real sites. For example, Ikotun et al. [14] reviewed many cases of geopolymer concrete pavement application. In India, although the paving action was performed experimentally in some areas, this work was successfully installed with geopolymer concrete [14]. In the case of asphalt, there are no clear alternative binders. Many cases involve adding additional materials into the asphalt binder, such as waste rubber powder [15], slag [16], fly ash [17], and so on. However, several cases of using recycled aggregate instead of natural aggregates have been confirmed. A 100% replacement of recycled aggregate showed a clear drawback in mechanical properties because recycled aggregate is a kind of second-hand material [18]. Thus, the inner matrix damage of aggregate itself cannot be avoided.

The use of by-products/recycled materials that take into account environmental concerns can bring about clear environmental benefits, but it is necessary to consider a large reduction in terms of usage performance. However, although sufficient performance will be secured by using existing materials, it is a major problem due to environmental factors. Considering these two paradoxical issues, this study confirmed that recycled plastic could be a good material for pavement. Plastic recycling is a well-known method for protecting the environment, and thermoplastic resin can make shapes easily. In fact, recycled plastic would show the same phenomena of strength reduction and decrease in durability, as the recycled plastic contains many kinds of pollution. Despite these issues, human beings should reduce the waste of plastics, and this approach of applying plastics as pavement materials can be a way of solving this problem. In prior studies, an approach was used to apply plastic waste into asphalt pavement with specific dosage ratios [19]. However, 100% plastic applications are a challenging work. It is not that there is no research on these challenges; the possibility was reviewed in a few studies. Iftikhar et al. [20] investigated the possibility of the application of waste plastic incorporating basalt fibers to be a pavement material. In the study of Iftikhar et al. [20], the problem was the security of compressive strength. Low-density polypropylene was investigated; however, the highest compressive strength was 21.5 MPa, even when containing basalt fiber [20]. Flexural strength was not measured. Another case by Thiam et al. [21] investigated high-density polypropylene and low-density polypropylene at the same time. In addition, Thiam et al. [21] added sand as a fine aggregate into the melted plastic paste and cured the specimens into mortars. Compressive and splitting tensile strength were measured. The compressive strength trend was the same as the study by Iftikhar et al. [20]—low values of around 16 MPa at the highest. However, splitting tensile strength showed a relatively higher value than compressive strength—around 4.6 MPa at the highest [21]. In the case of concrete, the typical range of splitting tensile strength of 16 MPa compressive strength is 0.96 to 2.4 MPa. Considering the compressive–tensile ratio of waste plastic, the potential of waste plastic to be a pavement material is enough if the compressive strength can be sufficiently obtained. To achieve this, adding magnesium hydroxide (Mg(OH)_2_) is the best answer. The reason is that Mg(OH)_2_ not only improves the brittleness and compressive strength balance of recycled plastics, but also enhances fire resistance and stiffness, which are critical for pavement durability.

Adding Mg(OH)_2_ makes plastic more brittle than pure plastic. In this state, the plastic with Mg(OH)_2_ may gain higher compressive strength than the pure state; however, this change makes the plastic lose its tensile properties. Flexural strength is considered the most important criterion for evaluating pavement materials, and a minimum flexural strength of 4.5 MPa is generally recommended. As the addition of Mg(OH)_2_ increases, the brittleness of the material also increases. Therefore, in this study, an experimental evaluation was conducted focusing on flexural strength, which is regarded as the most critical parameter. Nevertheless, few studies have systematically evaluated recycled plastic composites with Mg(OH)_2_, particularly with respect to flexural performance as a decisive parameter for pavement applications. This study aims to fill this research gap by clarifying both the material feasibility and the computational methodology for handling incomplete datasets.

As this study represents a new material challenge for road pavement, many issues inevitably arise. The addition of Mg(OH)_2_ to plastic introduces cementitious properties, which decrease the homogeneity of the matrix. Therefore, five specimens were tested for flexural strength, and the average value was calculated; however, the results were inconsistent. In some cases, missing data even occurred. This occurred due to unexpected specimen failure during flexural testing, such as cracking before full load-displacement data could be recorded. Missing data clearly affect the trend of data in material determination, one of the purposes of this study. In other words, it implies that incorrect material selection may be possible. Missing data must be analyzed even through estimation, and with the recent development of artificial intelligence, a good estimation method can be utilized: machine learning (ML). ML refers to a class of computational methods that enable systems to learn patterns from data and make predictions without being explicitly programmed. Its main advantages are the ability to capture nonlinear relationships, handle noisy or incomplete data, and provide robust estimations even under limited experimental conditions. There are many models of ML, like the estimation of missing data, such as tree-based models (decision tree, random forest) [22,23], boost models (XGBoost, CatBoost) [24,25], regressions (Gaussian process regression, nonlinear regression) [26,27], and neural network (NN) models [28,29,30]. These methods have been widely used so far, and the performance of the model has already been proven in many ways [31,32]. Due to the characteristics of the experiment, this study cannot obtain a large amount of experimental data for ML. Additionally, there is a clear weakness in using only a single model in consideration of these characteristics. Among the introduced models, it would be most effective to fuse a neural network and a tree or a boost model. However, merging different models, especially NNs merging a tree or a boost model, increases the computing cost and slows training. Although most ML studies indeed rely on large datasets, recent research has demonstrated that Bayesian-based neural networks are effective for small and noisy experimental datasets, since they explicitly model uncertainty. Therefore, this study applied a Bayesian long short-term memory (BLSTM) to ensure robust estimation despite the limited data size. Thus, this study utilized a fundamentally fused ML model—BLSTM. Already, a Bayesian NN (BNN) is actively used in time-series data analysis [33], numerical data training [34], prediction using small data groups [35], and so on. BNN originates from the basic NN architecture of artificial NN (ANN), and Bayesian algorithms are added to the ANN architecture. The differences between BNN and ANN are that BNN treats weights in training as probability distributions following Bayesian rules, unlike fixed calculated weights in ANN. Thus, BNN highlights the randomness of outputs and can reflect the uncertainty. Regarding data recovery, recent studies increasingly highlight generative approaches, with generative adversarial networks (GANs) most widely used, especially for image restoration and other high-dimensional reconstructions. Nevertheless, adversarial training is data-hungry and often unstable; robust performance typically requires large, diverse datasets [36]. Given our small, experimental DFSC time-series, GANs were deemed unsuitable here, so we employed a BLSTM to ensure robust estimation with quantified uncertainty. BLSTM is an advanced architecture compared to BNN because BNN contains the Bayesian theorem in ANN, but BLSTM contains the Bayesian theorem in LSTM, which is the recurrent architecture. The biggest difference between ANN and LSTM is the use of transferring data from hidden states. ANN trains with a basic process of forward propagation to backpropagation without previous hidden state calculations, but LSTM trains with a recurrent process of forward propagation to backpropagation with previous hidden state calculations. Thus, LSTM usually shows higher performance than ANN; it then can be expected that BLSTM will show higher performance than BNN.

Despite these prior efforts, there remains a clear research gap. It is hard to determine whether the existing works have systematically evaluated recycled plastic composites incorporating Mg(OH)_2_ with respect to flexural strength as the key performance criterion for pavement. Moreover, existing studies have not addressed how to manage missing experimental data, which can critically influence material selection. In addition, most ML applications in construction materials rely on thousands of samples, whereas small but high-quality datasets have been rarely explored. These limitations underscore the necessity of this study. BLSTM is combined with LSTM and the Bayesian theorem, but there are not only advantages. Harmonizing two different kinds of concepts is hard work. Since statistical weight update for data learning is performed at the same time, the required epoch value is basically high. Although it depends on the type of data, it is true that stochastic weight optimization in general requires significant computational steps. Thus, automatic stop systems or finding the best epoch works are essential in Bayesian-added NN architectures. The automatic stop system is widely used in practical industries where practicality is emphasized. However, this study was conducted with more focus on approaching this issue academically and finding the best epoch value.

This study is a kind of ML-based decision-making study, but it is a complex study that considers material aspects together. Therefore, the study was carried out in two steps. The first step is missing data estimation of the displacement–flexural stress curve (DFSC). This study investigated a total of nine cases (five specimens were tested per case) of plastic and Mg(OH)_2_ combination; six cases had missing data. Thus, missing data were estimated and completed the dataset. The second step is the tendency assessment of the dataset and the decision of the best mixture properties. The following sections describe the experimental procedures, data estimation methods, results, and conclusions in detail.

## 2. Materials and Methodologies

### 2.1. Materials and Flexural Strength Test

#### 2.1.1. Main Body Materials

This study utilized a total of three cases of a plastic main body. The first plastic was polypropylene (PP). PP is a representative thermoplastic polymer which is prepared through a polymerization reaction of a propylene monomer. The melting temperature is 160–170 °C; therefore, PP is easy to mold and process. A problem in the use of PP for the pavement material is that it has a low density of 0.9 ton/m^3^. Compared to the values for conventional pavement materials, such as concrete (2.5 ton/m^3^) and asphalt (2.4 ton/m^3^), this value is quite a low value for applying to pavement. It is even lower than the density of water of 1.0 ton/m^3^. For this reason, the specimens were prepared as composites with Mg(OH)_2_, since Mg(OH)_2_ has a density of 2.36 ton/m^3^.

The second plastic was polyethylene terephthalate (PET). PET is also a representative thermoplastic polyester manufactured through the condensation polymerization of ethylene glycol and terephthalic acid. PET is known to have excellent mechanical strength, heat resistance, chemical resistance, transparency, and workability. In general, tensile strength and flexural strength are high, and abrasion resistance is also excellent. The melting temperature is 250–260 °C, which makes it less deformable than PP at high temperatures. The density of PET is 1.4 ton/m^3^, higher than water; however, it is still lighter than concrete and asphalt.

The last plastic was OTHER resin. Plastics are distinguished by high-density polyethylene, low-density polyethylene, PP, polystyrene, polyvinyl chloride, and OTHER. OTHER means literally other; in this study, it is indicated with an abbreviation of OTH to distinguish it easily. OTH means that the plastic contains thermoplastic and thermosetting materials. This category includes a variety of special and highly functional plastics such as polycarbonate, polyurethane, acrylonitrile–butadiene–styrene, polyamide, polymethyl methacrylate, and polyether ether ketone. OTH resins have different chemical structures and properties, and in general there are many materials with excellent heat resistance, impact resistance, chemical resistance, and abrasion resistance. Polycarbonate has a melting point of about 230 °C, polyamide has a melting point of about 220–230 °C, acrylonitrile–butadiene–styrene has a melting point of about 105 °C, and polyether ether ketone has a melting point of about 340 °C. As such, OTH resin groups have various ranges in terms of thermal properties, durability, and mechanical performance, and are widely used in industries requiring high-functional materials such as electricity and electronics, automobiles, medical care, and aerospace. In this study, it was considered that the most suitable material for this study was polycarbonate-based OTH.

These three selected kinds of plastic bodies were prepared using recycled pellets, and the specimens were made using these pellets.

#### 2.1.2. Magnesium Hydroxide

**Figure 1:**
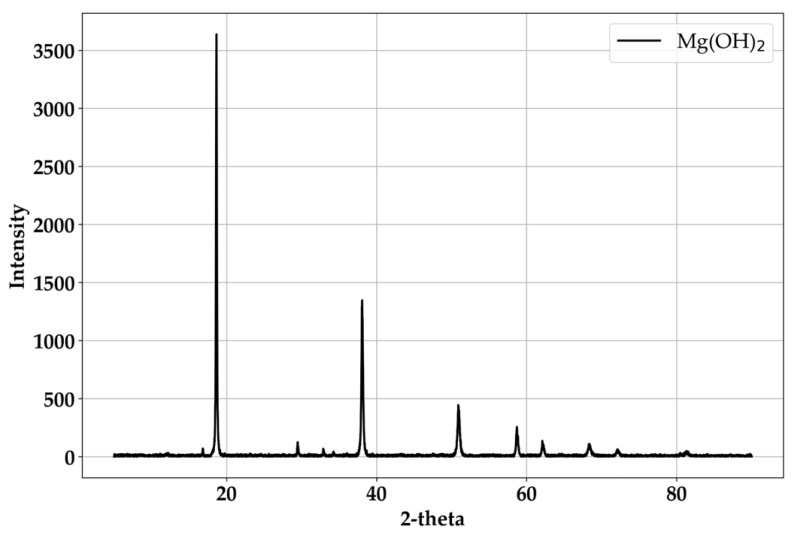
X-ray diffraction pattern of Mg(OH)_2_.

The composites considered in this study were intended to apply to road/highway pavement materials. Only the plastic body, its strain ratio, strength, and fire resistance are not suitable for application in pavement. It needs some brittleness, high fire resistance, and stiffness first. In particular, the plastics used in this study were all thermoplastic kinds; therefore, the inorganic material should have a chemical reaction with the fire–air–hydrogen parts in the matrix of plastics to prevent fast fire melting. Considering the conventional plastic industry and the target of this study, Mg(OH)_2_ was chosen as the best material. Applied Mg(OH)_2_ is a typically used material in many industries; Figure 1 indicates the used Mg(OH)_2_ in this study was the conventional material.

#### 2.1.3. Mix Properties and Specimen Details

Specimens for the flexural strength test were mixed at specific ratios. The main materials of specimen composition are plastic pellets and Mg(OH)_2_; however, there were many additives to instill functions. Usually, styrene–ethylene–butylene–styrene (SEBS) is used as an elastomer, which instills an elastic property in plastic. Another material was carbon black. As aforementioned, the purpose of this study was to use plastics to develop a novel pavement material; therefore, the color was important. Assuming that the pavement color is white, sunlight reflection makes driving difficult. The black color of asphalt makes driving easy, and the concrete pavement color is gray but not severe in the reflection of sunlight. However, typical colors of plastics are usually transparent white and light reflections; therefore, it was necessary to darken the color artificially. Hence, carbon black was selected and contained the additional advantage of ultraviolet light resistance. Table 1 contains the mixture properties of specimen production.

**Figure 2:**
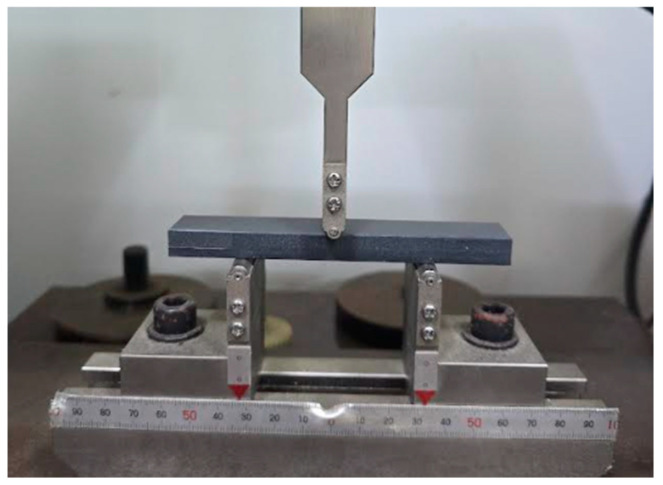
Flexural strength testing.

Following Table 1, five specimens were prepared in each case, and the dimensions of each specimen were 10 × 20 × 130 mm^3^. The span length was 65 mm. Originally, the standard (KS M ISO 178) [37] requires the sizes for measuring flexural strength of plastics of 4 × 10 × 80 mm^3^. However, to satisfy the civil engineering test standards as much as possible, we deliberately increased the specimen size, but this decision involved finding the middle points of the production conditions and test requirements between the plastic standard of KS M ISO 178 [37] and the cement mortar standard of ASTM C348 [38]. Figure 2 shows a flexural testing example.

### 2.2. Machine Learning for Missing Data Estimation

**Figure 3:**
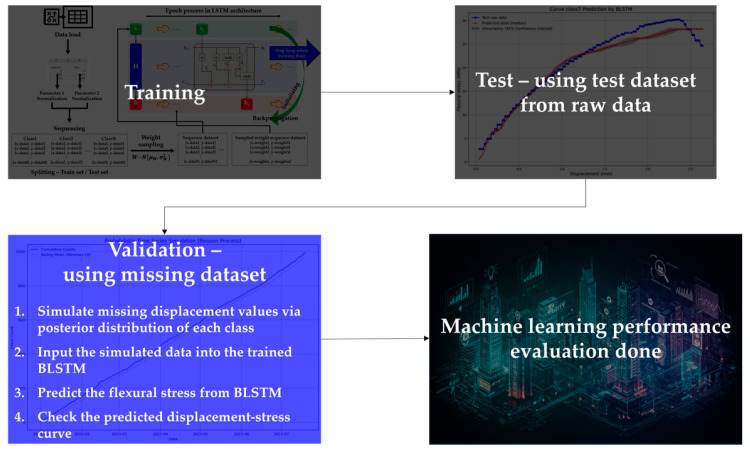
Total process of BLSTM ML for missing curve data estimation.

In this study of ML, four steps to evaluate BLSTM performance were carried out. Figure 3 exhibits the total process of BLSTM ML, but it was a little different from typical ML studies. Prior ML studies usually used enormous amounts of data; however, this study performed ML with limited amounts of data. Thus, this study decided to utilize BLSTM because Bayesian-combined models have advantages in small data groups.

#### 2.2.1. Data Collection

**Figure 4:**
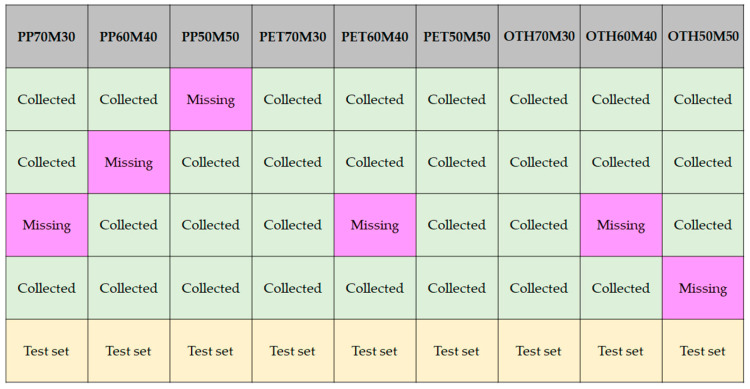
Data collection status.

From the experiment in Figure 2 involving the flexural strength test, DFSC data were collected. During the test, some missing data were broken out. Figure 4 summarizes the data collection state of this study.

Three data cases of PET70M30, PET50M50, and OTH70M30 were fully collected; however, the rest of the cases have one missing dataset each. Five specimens were tested in each class; therefore, one data case in each class was left as the test dataset. The green-colored datasets were used as the training dataset, and the missing values were the target of estimation–validation. The DFSC details are arranged in Section 3.

#### 2.2.2. Bayesian Statistics

BLSTM is merged with the theorem of Bayesian statistics (BST); therefore, it is essential to understand the concept of BST. Traditional statistical approaches focus on finding averages and standard deviations. On the other hand, BST considers two states of probabilities: the pre-state probability of prior probabilities/distributions and the post-state probability of posterior probabilities/distributions. In addition, posterior state is a kind of estimation work; therefore, the prior probability from real data is important because this state is continuously updated. BST can be expressed as Equation (1):

(1) $$p\left(\alpha |\beta \right)=\frac{p\left(\beta |\alpha \right)p(\alpha )}{p(\beta )}$$

where p(α) is the prior probability, $p\left(\beta |\alpha \right)$ is the likelihood, p(β) is the marginal probability, and $p\left(\alpha |\beta \right)$ is the posterior probability. Likelihood is affected by the prior probability; therefore, the most core probability is prior. Due to this relationship, the marginal probability is usually considered as a constant. Hence, BST is typically approached by the simplified state of $p\left(\alpha |\beta \right)\propto p\left(\beta |\alpha \right)p(\alpha )$ when finding the mean/variance of posterior probability. Equation (1) looks like it contains a simple method; however, it is a powerful statistical methodology, especially in small data groups, because the posterior state is derived by the data updating—therefore, estimation quality is typically high [39].

#### 2.2.3. Bayesian LSTM Training Process

**Figure 5:**
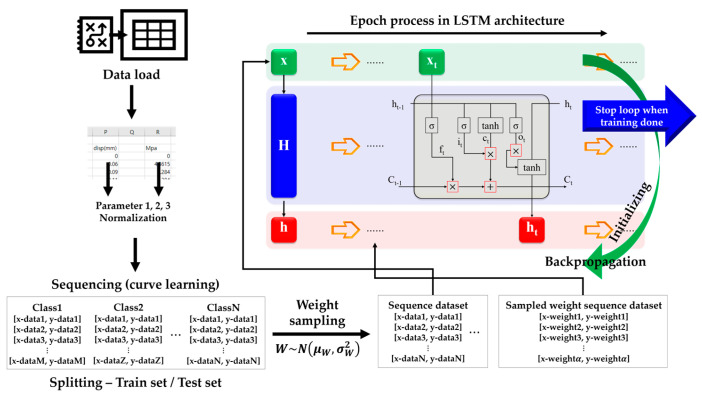
BLSTM training process.

From the flexural strength test, only displacement and stress were measured. With insufficient amounts of parameters or without clear representative parameters, ML performance could not be secured. Due to the limits of data gathering from the experiments, setting one more representative parameter was decided. The ratio of displacement/stress was checked, and it was confirmed that this ratio reflected the data behavior well. Thus, the displacement/stress ratio, displacement, and flexural stress were sequenced to train the BLSTM. The BLSTM training process is indicated in Figure 5.

**Figure 6:**
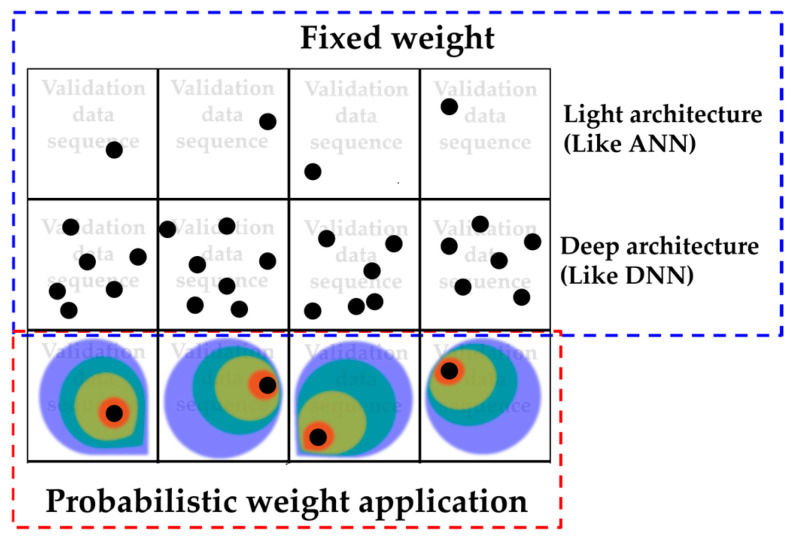
A schematic example of weight application difference.

A remarkable characteristic of BLSTM is weight sampling using the BST theorem. At first, randomly sampled weights are applied in a very early step of the epoch process; however, the weight distributions are changed by backpropagation from the LSTM architecture. Other research fields, such as languages, physics, and so on, train NN models with sequence data. Civil engineering fields may not be familiar with such data sequencing. However, it is a frequently selected method for learning the data behavior itself in various fields and is commonly used. On the other hand, sampled weight applications are different stories. Sequence data learning is conventionally performed; however, applications of probabilistic weights are hard to imagine. Simply, fixed weights may miss many parts when weights are applied to the validation data, but weight application in the state of probability distributions can cover many parts, as shown in Figure 6.

Figure 6 is a simple example of how different the application of weights is in conventional and probabilistic cases. In the case of the fixed weight of Figure 6, only black dots occupy their small point of area. This means that, when applied to the data at a fixed value, the prediction proceeds only for a fixed case without consideration of the considerable feasible range. However, it can be seen that the application of probabilistic weight covers a wide range of areas with feasible probability. In other words, it is possible to provide a feasible range for all predictions together. This is a significant merit to the prediction; therefore, this part can work advantageously for small data groups.

#### 2.2.4. Best Epoch

BLSTM, which is an advanced model of BNN, needs more epochs than conventional NNs. Due to this drawback, finding the best epoch value is essential if the automatic stop methodology is not applied. This study evaluated training loss, internal training R^2^ value per epoch, and weight norm (WN) during the train period. Training loss and R^2^ are expected to show an exponential or log shape graph; however, we are not able to expect how the behavior of WN will come out. When sufficient fluctuations occur, analysis of WN will be easy. However, there is a possibility that smooth continuous behavior occurs. If this hard state comes out, an additional method is required to analyze the WN. Therefore, this study considered applying fast Fourier transform (FFT). The start of FFT is a discrete Fourier transform, which can be expressed in Equation (2):

(2) $$X(k)=\sum_{n=0}^{N-1}x(n)e^{-ikn\frac{2\pi }{N}}$$

where x(n) is the domain signal (length N), X(k) is frequency–domain representation (complex spectrum), i is an imaginary unit ($i^{2}=-1$), $e^{-ikn\frac{2\pi }{N}}$ is the basis Fourier term (rotating phasor), and k=0, 1, 2, 3, ⋯, N−1. However, the state of Equation (2) takes a long time; therefore, an efficient way is derived—this is the FFT. FFT splits Equation (2) into two phases, an even phase of 2r and odd phase of 2r + 1, as shown in Equation (3).

(3) $$X\left(k\right)=\sum_{r=0}^{\frac{N}{2}-1}x\left(2r\right)e^{-ik\left(2r\right)\frac{2\pi }{N}}+\sum_{r=0}^{\frac{N}{2}-1}x\left(2r+1\right)e^{-ik\left(2r+1\right)\frac{2\pi }{N}}$$

In summary, Equation (3) can be expressed simply as $X\left(k\right)=E(k)+O(k)$, where E(k) is the even term and O(k) is the odd term. To efficiently analyze the WN for finding the best epoch, the WN will be differentiated up to the second state because the complex vibration can appear at least in the second differential state. After finding the best value of epochs, training was performed at the best epoch value again.

#### 2.2.5. Test and Validation

In Figure 4, test sets were set to one case per class. In the test, we evaluated how the trained model followed the tendency of the DFSC data. To move to the validation work, the average test R^2^ value should be greater than 0.8. It is typically considered that R^2^ values above 0.8 are highly reliable, between 0.7 and 0.8 means reliable, between 0.6 and 0.7 means reliable but less reliable than the 0.7 case, and less than 0.6 means less reliable [27]. Thus, the criteria for R^2^ values were set to be above 0.8.

At the validation stage, displacement/stress ratio and displacement were simulated for the missing cases, and BLSTM estimated the flexural stress. In this process, the BLSTM was trained with three sequential inputs: displacement, flexural stress, and the displacement/stress ratio. The model output was the predicted flexural stress values for the missing segments of the curves. By propagating hidden states across the sequence, the BLSTM was able to capture correlations between available and missing data points rather than relying on a simple input–output mapping. This mechanism ensured that the estimation was consistent with the overall curve behavior and preserved the physical response of material trends. After estimation, a direct comparison with training datasets in each case was performed.

## 3. Results and Discussion

### 3.1. Flexural Strength Test Results

**Figure 7:**
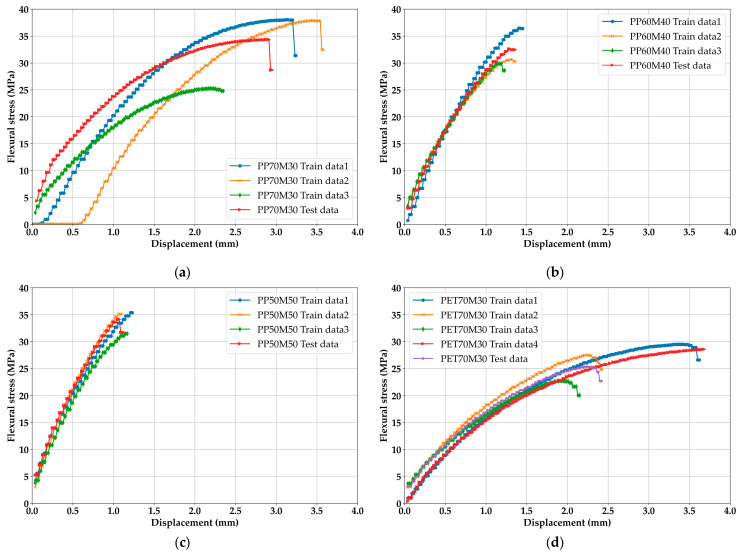

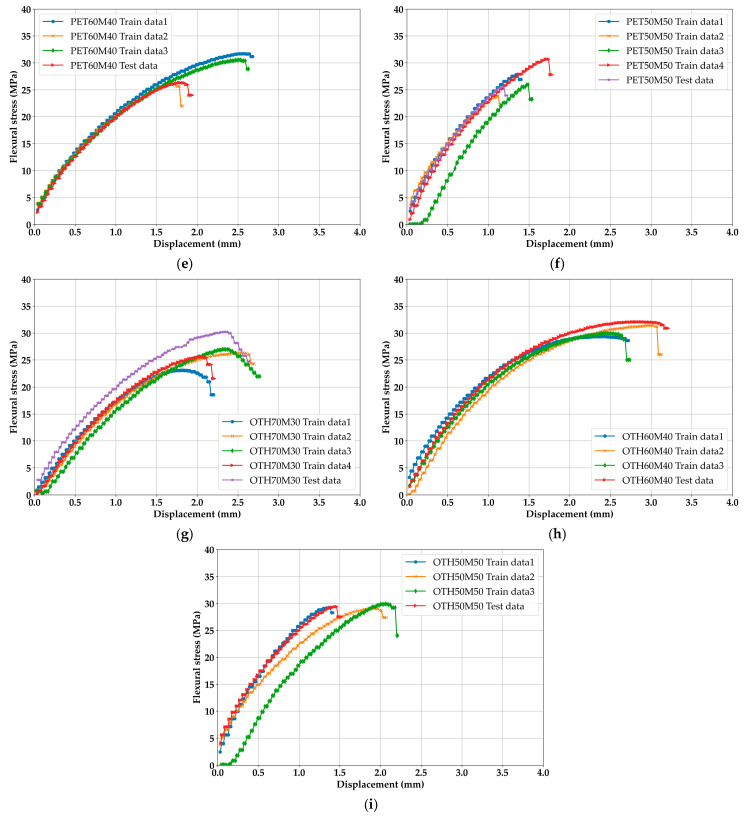
DFSC results: (**a**) PP70M30, (**b**) PP60M40, (**c**) PP50M50, (**d**) PET70M30, (**e**) PET60M40, (**f**) PET50M50, (**g**) OTH70M30, (**h**) OTH60M40, (**i**) OTH50M50.

Figure 7 contains the results of the DFSC with all data. Compared to PP, PET and OTH showed stronger ductility properties, even when Mg(OH)_2_ was added. PP showed an immediate decrease in ductility because Mg(OH)_2_ increases the brittleness. In practical, displacement exceeding 1 mm tends to be similar to that of organic-fiber-reinforced concrete in the flexural test. In other words, the displacements of Figure 7a,b can be considered as large displacements in civil engineering structures [40]. Comprehensively, this trend of PP is very consistent with the part intended in this study. In particular, in Figure 7b,c the deviation between data decreases due to the increase in brittleness, so when interpreted as a road pavement material, it can be regarded as the most suitable material. Small deviations in their own case data, such as in Figure 7d,e,g,h, can confirm this; however, large elongation can be a problem when these materials are significantly considered for further experiments. The reason why the increase in plastic brittleness is important is that the increase in brittleness leads to an increase in compressive strength. Plastic is a material that usually has a 1:1 corresponding compressive strength and tensile strength, and, in some cases, the flexural strength is greater than the compressive strength [41]. If brittleness increases by the addition of Mg(OH)_2_, the expected compressive strength can be inferred through flexural strength, and the high flexural strength shown in Figure 7b,c can mean securing high compressive strength. According to Figure 7, at least the flexural strength criteria for road pavement are not a significant problem.

**Figure 8:**
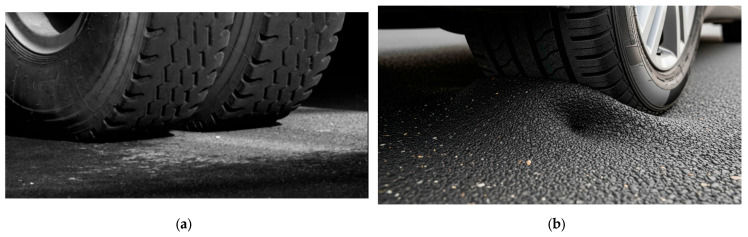
Explanation of rigid pavement and high-ductility pavement; (**a**) rigid state pavement [42], (**b**) an expected high-ductility pavement bearing the weight of vehicle.

In all cases, the flexural strength achieved a level of 30 MPa, but there are concerns about the aforementioned ductility. The reason why reducing ductility is important is explained with Figure 8.

Figure 8 further illustrates the practical significance of material behavior in pavement applications. As shown in Figure 8a, rigid pavements can effectively distribute vehicular loads with minimal local deformation, which is essential for long-term serviceability. In contrast, high-ductility pavements, represented in Figure 8b, undergo excessive deformation under wheel loading, which can eventually lead to rutting or fatigue cracking. PET- and OTH-based composites exhibited ductile characteristics similar to Figure 8b, whereas PP + Mg(OH)_2_ composites showed controlled brittleness that corresponds to the rigid-like response in Figure 8a. Therefore, achieving an appropriate level of brittleness through Mg(OH)_2_ addition is critical for the development of recycled plastic composites suitable for pavement applications.

This study is a basic study for material selection, and experiments other than flexural strength will be conducted in further studies. In future studies, the amount of Mg(OH)_2_ input is planned to be increased, and, in this case, unlike PP, PET and OTH may pose the concern of lowering the strength, as in the trend of DFSC. Unlike PPs close to 40 MPa, PET and OTH have strengths near 30 MPa. Considering that it is a recycled plastic, it cannot be guaranteed to maintain its strength in further studies.

However, six cases have missing data; therefore, debating the exact trends does not make sense. Clearly, estimations in missing data will appear in the acceptable/possible ranges through BLSTM. Hence, analyzing a more exact material selection should be performed after BLSTM estimation.

### 3.2. Training Results of BLSTM

**Figure 9:**
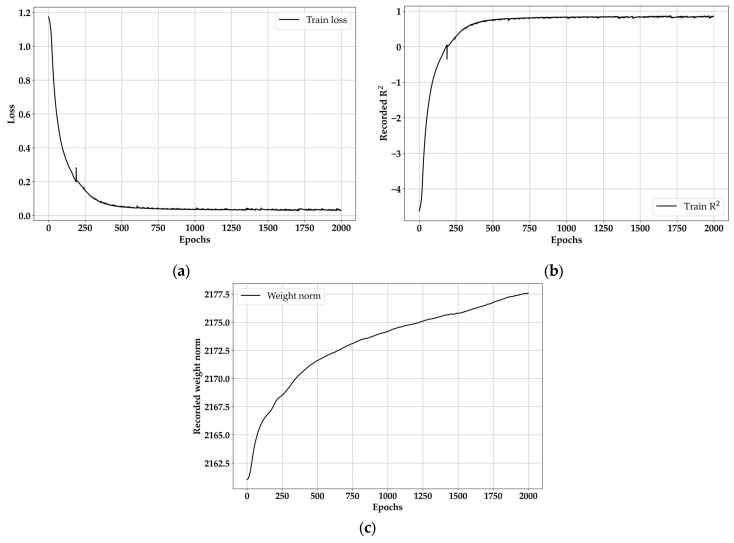
BLSTM training results: (**a**) training loss, (**b**) recorded training R^2^, (**c**) recorded training weight norm.

In order to find the best epoch value in advance, the training epoch value was set to 2000. As mentioned before, Bayesian merged models require a large number of epochs because of the weight harmonizing. Thus, a randomly chosen number of epochs was set as 2000, and the results of training of BLSTM are exhibited in Figure 9.

**Figure 10:**
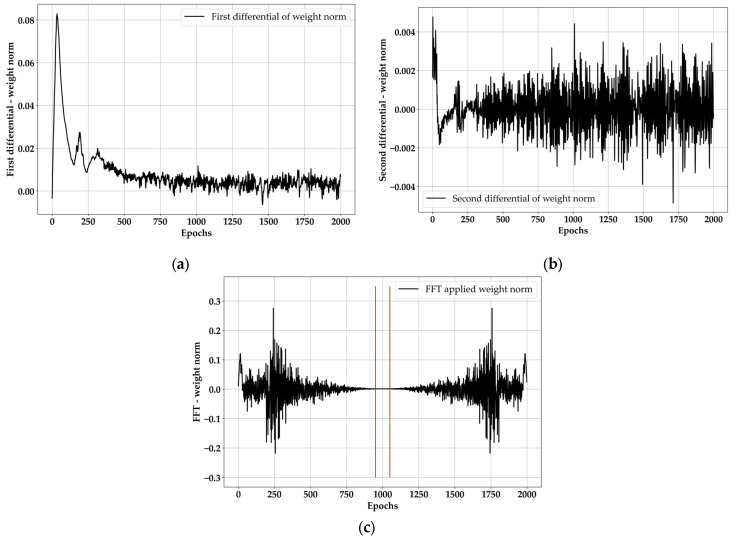
Differential and FFT application results: (**a**) first differential of WN, (**b**) second differential of WN, (**c**) FFT results through second differential of WN.

The results for loss and R^2^ behavior in Figure 9a,b are consistent with general NN training. Here, the behavior of Figure 9a,b will appear within 500 epochs in the case of general NN models [43], but BLSTM is a heavier model than BNN, and the weight harmonizing appeared strongly, showing a stable tendency after 750 epochs. However, it is not possible to evaluate the best epoch value with Figure 9a,b, since they are significantly smooth after 750 epochs. That does not mean that Figure 9c shows a clear tendency. Like loss and R^2^, it only increases smoothly with the progress of the epoch. Therefore, a second differentiation and FFT were performed on WN, and the results are shown in Figure 10.

It should be acceptable to apply the FFT method in the first differential state; however, the vibration before 380 epochs was significantly weak, and the peaks were not stable. Thus, the FFT method could not be applied to the first differential state. The second differential state showed more stable vibrations, which are like the ultrasonic pulse vibrations [44]. There is a large variation in the early epoch steps before 250, but it was enough to apply the FFT. According to Figure 10c, a flat area exists between 940 and 1050 epochs. This means that 940 to 1050 epochs is the range for the best epoch nominee. At this point, the continuous increase in Figure 9c is not meaningless. When evaluated together with Figure 9c in the state that the nominee groups are organized, the best epoch is 1050. The reason is that the weight norm means the expression level of the model [45], so even if the loss and training R^2^ show a flat behavior after 750 epochs, the expression level continued to increase.

As a result, the best epoch value was determined to be 1050. With this best epoch, the training was performed again, and the test was carried out directly.

### 3.3. Test Results of ML

**Figure 11:**
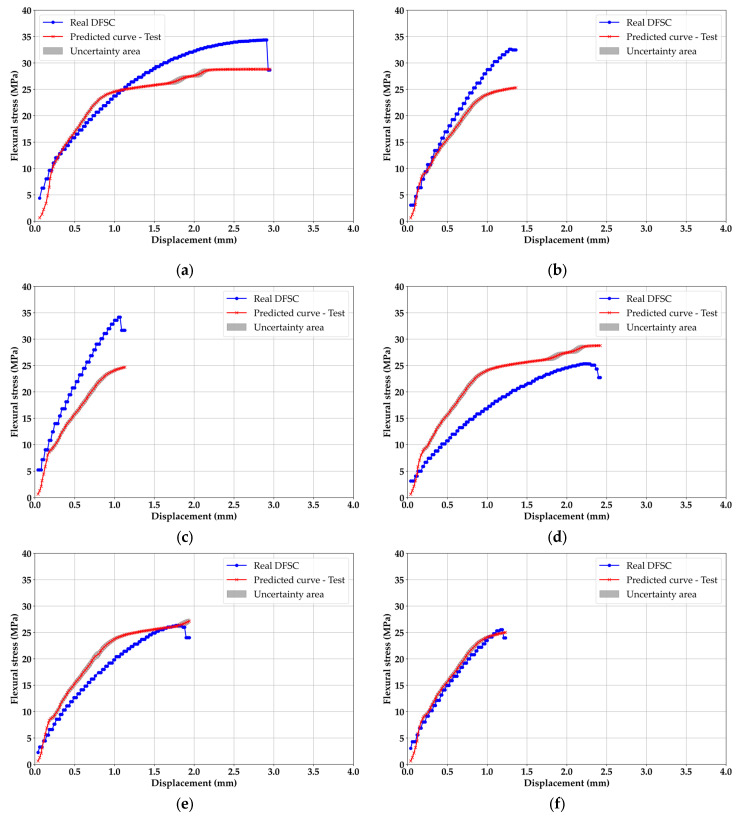

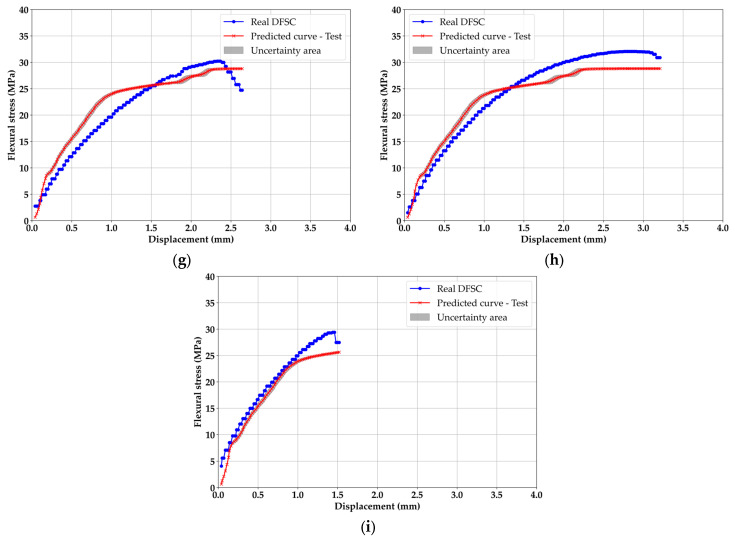
ML test results: (**a**) PP70M30, (**b**) PP60M40, (**c**) PP50M50, (**d**) PET70M30, (**e**) PET60M40, (**f**) PET50M50, (**g**) OTH70M30, (**h**) OTH60M40, (**i**) OTH50M50.

Figure 11 contains the results of the ML test in this study. The outcomes of test results must be possible ranges. In this respect, the test results of Figure 11b,c become ambiguous in this state. The other cases of test results showed a high fitting tendency; however, only Figure 11b,c did not fit well. The evaluation of these two cases in Figure 7 was the most positive, but, on the contrary, the test results for these two cases in Figure 11 were the worst. Even the deviation was the highest generated. This phenomenon is considered as sequential data training; therefore, the other model behavior may affect Figure 11b,c. BLSTM trained the curves themselves in each case; the weights must contain the probabilistic information in the posterior state. In terms of the BLSTM mechanism, it is highly possible that trained weights have universal probability distributions [46]. With this interpretation, the deviation between Figure 11b,c can be explained. This is the result obtained by learning all cases at the same time for the efficiency of learning. Since the experiment was conducted through a relatively heterogeneous matrix by adding Mg(OH)_2_ to plastic, this study concluded that these two large deviations could also occur, and it was decided to use the current trained BLSTM for the final validation. In addition, the average test R^2^ value was 0.8018.

### 3.4. Validation Results of ML

**Figure 12:**
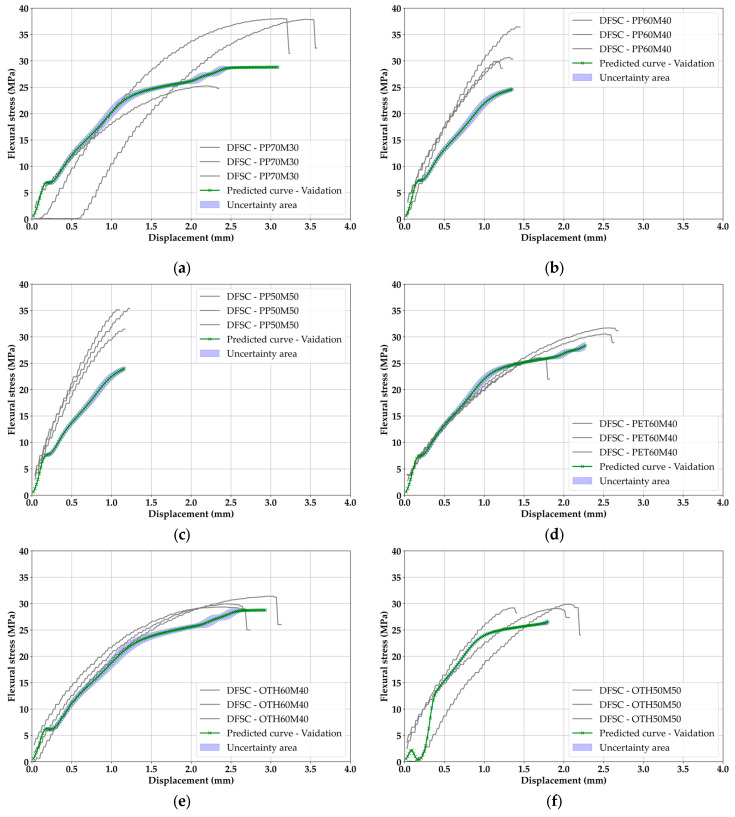
Validation results: (**a**) PP70M30, (**b**) PP60M40, (**c**) PP50M50, (**d**) PET60M40, (**e**) OTH60M40, (**f**) OTH50M50.

Figure 12 exhibits the results of validation in this study. Predicted curves were considered one of the DFSC data, since the original purpose of validation was to reinforce the missing data to fill the gaps. All the validation results were similarly derived but different in detail; however, Figure 12f showed different behavior from Figure 11i in the test. In an early stage of displacement between 0 to 0.25 mm, a weak camber was confirmed, and it fell back to 0 and showed a tendency to rapidly rise again after 0.25 mm. This is a trend that could not be confirmed in other cases of tests and validation. Unlike training and tests, which were accompanied by data in the learning process, it is a result obtained by pure simulation without input data, and a positive evaluation cannot be given for OTH50M50.

Despite the case of OTH50M50, the average validation R^2^ was derived to be 0.7618. Most of the trends of the upward curve followed closely, and, in addition, some cases settled completely within the range of existing data. In general, when R^2^ is measured in tests and validation, the performance in validation often shows a large difference [47]. However, the results in this study are very small, as the difference in R^2^ between test and validation is 0.04. This is very encouraging, and it can be interpreted that the probabilistic approach, to some extent, has been supplemented to narrow the difference [48]. 

Table 2 shows noticeable dispersion across metrics, with the PP series (PP70M30–PP50M50) having higher RMSE/MAE and MAPE than PET60M40 or OTH mixes. Importantly, these values are relative deviations from the class mean and percentile baselines rather than absolute errors, so they can appear larger for curves with higher peaks. When cross-checked with Figure 12 and a direct visual inspection of the DFSCs, the PP composites still display consistent rigid-like shapes and peak ranges, which supports their suitability for pavement. Moreover, the model fit (validation average R^2^ = 0.7618) indicates that the predictive trends are captured effectively despite the observed spread. Hence, the PP errors should be interpreted as acceptable for decision-making in this small-sample experimental context.

Comprehensively, the trained BLSTM in this study is in the range of highly reliable and reliable—it may be able to be considered as a semi-high reliable state. Thus, estimated curves can be trusted; therefore, the final decision for a novel smart mobility pavement material was made with this estimated data.

### 3.5. Main Resin Determination

Table 3 is the decision-making process for the best resin for potential pavement material selection. Based on this study, a total of five categories are arranged: strength, reflection of brittleness, test data deviation of ML, test/estimation stability of ML, and potential for pavement application in our subjective opinion. For each item, the degree of quality was expressed in color, and the degree of color for each step was indicated by footnotes. The potential terms in Table 3 also considered the melting points and the other properties of each resin.

According to the evaluation in Table 3, PP is the resin that has the highest potential to be a pavement material; PET is the next, and OTH is concluded to be not suitable for pavement material. In the case of decision in OTH, the most critical points were the reflection of brittleness and the potential term in Table 3. According to ML results, OTH showed the most stable results, and this study decided to believe that the estimated data is the measured data. Therefore, OTH can be investigated in further study along with PP; however, the brittleness reflection is important, and the potential points of its own properties are not suitable. On the other hand, it was concluded that PET deserves to be re-examined with PP in further studies. When further study is carried out, the plan will change depending on the condition; however, PET has nevertheless proven to be sufficiently worth investigating if brittleness control is performed effectively.

The resin determination in Table 3 can also be interpreted in light of the pavement responses illustrated in Figure 8. PET- and OTH-based composites, although achieving the minimum flexural strength requirement, displayed relatively higher ductility. This behavior corresponds to the case shown in Figure 8b, where excessive local deformation occurs under wheel loading. Such deformation may lead to rutting or long-term instability, suggesting that PET should be reconsidered only if its brittleness can be effectively controlled. In contrast, OTH resins showed stable ML estimation results but lacked sufficient brittleness reflection, which again corresponds to the ductile response in Figure 8b. Therefore, despite some positive aspects, it concluded that OTH was unsuitable for pavement applications. On the other hand, PP composites with Mg(OH)_2_ exhibited controlled brittleness closer to the rigid response illustrated in Figure 8a, supporting their selection as the most promising candidate.

## 4. Conclusions

This study fabricated composites by incorporating Mg(OH)_2_ into recycled plastics and comprehensively evaluated their potential as next-generation pavement materials. For this purpose, three types of recycled plastics, including PP, PET, and OTH resins, were used to prepare a total of nine specimen combinations, and their mechanical behavior and flexural performance were analyzed through DFSC. Missing data generated during the experimental process were complemented by applying a BLSTM ML with a strong probabilistic prediction capability, enabling reliable curve reconstruction and completion of the dataset. The detailed conclusions are as follows:

1. The flexural strength results indicated that all combinations achieved a minimum flexural strength of 30 MPa. The incorporation of Mg(OH)_2_ is expected to increase the compressive strength due to the accompanying rise in material brittleness. Among the tested specimens, the PP-based composites exhibited the highest performance, with approximately 40 MPa of flexural strength, whereas the PET- and OTH-based composites showed values around 30 MPa.
2. However, the reflection of brittleness showed that PP was the best, and PET and OTH were not sufficient. Assuming that the materials of this study are applied to road pavement, brittleness is emphasized because the high-ductility part greatly reduces the performance of road pavement in the future. Thus, the evaluation of PET and OTH in this area is conservative.
3. BLSTM model training identified the optimal epoch as 1050. The average R^2^ values of 0.8018 for the test set and 0.7618 for the validation set confirmed that the model achieved a reliable prediction level. Furthermore, the validation performance showed only a minor decrease compared with the test performance, indicating that the probabilistic BLSTM approach is effective even for small-scale datasets with limited experimental observations. This result demonstrates the capability of the trained model to reconstruct missing DFSC while maintaining prediction reliability.
4. The material selection results based on Table 3 indicate that PP is the most suitable candidate, as it exhibits optimal flexural strength and brittleness characteristics, making it highly promising for pavement applications. PET warrants further investigation, particularly if its brittleness can be effectively controlled in future studies. In contrast, OTH resin, despite showing stable behavior in ML estimations, was considered to be unsuitable due to its insufficient brittleness response and limited potential for practical pavement application.

Overall, the results indicate that Mg(OH)_2_ added PP exhibits the most promising balance of flexural strength and brittleness for pavement applications, PET shows potential if its ductility can be controlled, and OTH is unsuitable. The BLSTM model successfully addressed missing data and ensured reliable evaluation despite the small dataset, providing a robust basis for material determination. In addition to further steps, other models to evaluate the performance of plastic composites can be considered, such as DeepLab [49] or EfficientNet [50].

## Figures and Tables

**Table 1:** Mixture properties.

Weight Percent (%)				
**Case**	Resins	Mg(OH)_2_	SEBS	Carbon Black
PP70M30	66.5	28.5	3	2
PP60M40	57	38		
PP50M50	47.5	47.5		
PET70M30	66.5	28.5		
PET60M40	57	38		
PET50M50	47.5	47.5		
OTH70M30	66.5	28.5		
OTH60M40	57	38		
OTH50M50	47.5	47.5		

**Table 2:** Error indices according to the ML validation results.

Specimens	RMSE	MAE	MSE	MAPE (%)
**PP70M30**	3.2464	2.9583	10.5391	28.0063
**PP60M40**	6.9086	6.2744	47.7290	29.7634
**PP50M50**	8.6552	7.9614	74.9132	37.9315
**PET60M40**	1.2373	1.1365	1.5310	7.9076
**OTH60M40**	2.4448	2.2233	5.9768	11.8896
**OTH50M50**	3.7010	2.9760	13.6954	24.1213

**Table 3:** Decision-making process for the best resin for potential pavement material.

Resins	Strength ^1^	Reflection of Brittleness ^2^	Test Data Deviation (Including Estimation) ^3^	Test/Estimation Stability ^4^	Potential of Pavement Application ^5^
**PP**	Best	Best	Large	Normal	Suitable
**PET**	Good	Bad	Median	Normal	Needs more investigation
**OTH**	Normal	Worst	Small	Good	Not suitable

^1, 2, 4^ Best–Good–Normal–Bad–Worst. ^3^ Smallest–Small–Median–Large–Largest. ^5^ Suitable–Needs more investigation–Not suitable.

## Data Availability

The original contributions presented in this study are included in the article. Further inquiries can be directed to the corresponding author.

## Nomenclature

**Symbol**	**Definition**	**Unit/Note**
p(α|β)	Posterior probability	-
p(β|α)	Likelihood	-
p(α)	Prior probability	-
p(β)	Marginal	-
x(n)	Time/sample-domain signal	Length N sequence
X(k)	Frequency-domain representation (DFT of x)	Complex spectrum
N	Signal length	-
n	Time/sample index	0, …, N−1
k	Frequency-bin index	0, …, N−1
r	Index for even/odd split	0, …, N/2−1
i	Imaginary unit	$i^{2}=-1$
e	Complex exponential	-
π	Circumference	3.14
E(k)	Even term in FFT split	-
O(k)	Odd term in FFT split	-
**Abbreviations**	**Definition**	**Unit/Note**
DFSC	Displacement–flexural stress curve	-
R^2^	Determination coefficient	-
WN	Weight norm	
DFT	Discrete Fourier transform	-
ML	Machine learning	-
ANN	Artificial neural network	-
BNN	Bayesian neural network	-
LSTM	Long short-term memory	-
BST	Bayesian statistics	-
PP	Polypropylene	Thermoplastic
PET	Polyethylene terephthalate	Thermoplastic polyester
OTH	Other resins	Category label
Mg(OH)_2_	Magnesium hydroxide	-
SEBS	Styrene–ethylene–butylene–styrene	Elastomer
GAN	Generative adversarial network	
RMSE	Root mean squared error	
MAE	Mean absolute error	
MSE	Mean squared error	
MAPE	Mean absolute percentage error	
